# Views from ‘crabworld’: the spatial distribution of light in a tropical mudflat

**DOI:** 10.1007/s00359-023-01653-7

**Published:** 2023-07-17

**Authors:** Jochen Zeil

**Affiliations:** Research School of Biology, Australian National University, P.O. Box 475, Canberra, ACT 2601 Australia

**Keywords:** Spectrographic imaging, Natural scene, Environmental light, Polarized light, Mudflat, Fiddler crabs

## Abstract

Natural scene analysis has been extensively used to understand how the invariant structure of the visual environment may have shaped biological image processing strategies. This paper deals with four crucial, but hitherto largely neglected aspects of natural scenes: (1) the viewpoint of specific animals; (2) the fact that image statistics are not independent of the position within the visual field; (3) the influence of the direction of illumination on luminance, spectral and polarization contrast in a scene; and (4) the biologically relevant information content of natural scenes. To address these issues, I recorded the spatial distribution of light in a tropical mudflat with a spectrographic imager equipped with a polarizing filter in an attempt to describe quantitatively the visual environment of fiddler crabs. The environment viewed by the crabs has a distinct structure. Depending on the position of the sun, the luminance, the spectral composition, and the polarization characteristics of horizontal light distribution are not uniform. This is true for both skylight and for reflections from the mudflat surface. The high-contrast feature of the line of horizon dominates the vertical distribution of light and is a discontinuity in terms of luminance, spectral distribution and of image statistics. On a clear day, skylight intensity increases towards the horizon due to multiple scattering, and its spectral composition increasingly resembles that of sunlight. Sky-substratum contrast is highest at short wavelengths. I discuss the consequences of this extreme example of the topography of vision for extracting biologically relevant information from natural scenes.

## Introduction

Vision has evolved in the concrete situation of a specific animal’s environment and behavioral setting. The general properties of visual information processing mechanisms thus reflect both the invariant spatio-temporal aspects of vision in the natural world and the properties of specific visual habitats. The great diversity of visual habitats is witnessed by the ubiquitous optical, retinal, and neural specializations found in biological vision systems (for reviews see Walls 1942; Hughes 1977; Zeil et al. 1989; Nalbach 1990; Archer et al. 1999; Land and Nilsson 2001; Eckert and Zeil 2001; Baden et al. 2020). On a certain level, visual systems are similar across species, yet on another, none resembles the other. We thus face the task to discriminate between the general and the specific constraints of visual processing, and the selective forces that have shaped the evolution of its diverse designs.

The general constraints on vision are best understood at the level of optical processing where the conflicting demands on resolution and absolute light sensitivity have been identified in great detail (for reviews see Warrant and McIntyre 1993; Land and Nilsson 2001). The problem of how neural signal processing copes with the limited bandwidth of neurons in the presence of large variations in signal intensity, of environmental, and of neuronal noise has also been extensively addressed (reviewed by Laughlin 1990; van Hateren 1992, 1993; Warrant 1999). Information theory has been used to understand the receptive field properties of interneurons in the early visual pathway and the evolution of color vision in the light of natural image statistics (e.g. Barlow 1961; Field 1987; Atick 1992; Ruderman 1994; Dong and Atick 1995; van der Schaaf and van Hateren 1996; Burton 2000; Chiao et al. 2000; see Simoncelli and Olshausen 2001 for review). The analysis of images taken in various natural environments has shown that the distribution of spatial frequencies, their power spectrum, can be described by a function of the type c/f^n^, whereby c is a constant, f is the spatial frequency and n typically lies between 1.5 and 3 (for a review see van der Schaaf and van Hateren 1996). This invariant spatial property of the visual world forms the basis for optimal strategies of redundancy reduction and predictive coding in visual information processing (e.g. Srinivasan et al. 1982; Rao and Ballard 1999).

However, when invoking theory of information processing to understand the design of sensory and neural filters, assumptions need to be made about the constraints operating on a given system, and about the tasks that system has evolved to cope with (e.g. Baddeley 2000; Eckert and Zeil 2001). For instance, it is always assumed that all aspects of an image are equally important, or in other words that all image contents need to be transmitted by the early stages of visual processing. This may not always be the case: the visual systems of many animals appear to be designed to act as filters matched to the relevant information content of the animal’s visual world, in which only a limited number of events and their specific visual signatures are significant (Wehner 1987). This does not mean that the general properties of natural scenes did not influence the design of signal processing mechanisms. First, the invariant spatial and spectral statistics of natural scenes will be reflected in the design of signal processing pathways concerned with tasks like the control of locomotion and orientation (optomotor stabilisation), or the ambient light measurements for circadian rhythm entrainment, contrast gain control, or light and dark adaptation. The evolution of early visual processing will thus have been influenced by the requirements of tasks the solution of which profit from making use of the properties of the whole distribution of contrast, color, or contour orientation in a scene. Second, at the same time, the statistics of natural scenes are relevant as a background for visual recognition tasks, i.e. the detection, identification, and discrimination of targets in terms of luminance, spectral, spatial frequency, orientation, and motion contrast. Food sources (prey, flowers), predators, conspecifics, communication signals, landmarks, compass direction, significant places, all have specific visual signatures which animals have evolved to extract efficiently from natural scenes. Third, in addition, most animals view the world from a certain perspective and with a carefully controlled orientation of their visual system (e.g. Nalbach 1990; Hengstenberg 1993), so that the spatial, spectral, polarization, orientation, and motion statistics are likely to differ predictably in different parts of the visual field. This is witnessed by the fact that most visual systems do not sample the world in a uniform fashion like a video camera: the eyes of animals all show varying degrees of regionalization of optical and neural processing across the visual field (e.g. Hughes 1977; Nalbach and Nalbach 1987; Land 1999a). Last but not least, it is important to note that behavior structures the flow of visual signals at the retina (e.g. Eckert and Buchsbaum 1993; Land and Collett 1997; O’Carroll et al. 1996, 1997; Eckert and Zeil 2001; Zeil et al. 2008). The particular behavioral repertoire of an animal therefore has a critical influence on signal statistics at the retina and on the subsequent neural mechanisms of extracting relevant information. In some instances it has indeed been shown that behavior is specifically tailored to aid visual information processing (e.g. Land and Collett 1997; Eckert and Zeil 2001; Kral 2003; Zeil et al. 2008; Egelhaaf 2023).

Taking all these facts of visual ecology into account, there is thus a great need to accurately describe the natural operating conditions of visual systems, with the goal to understand image processing requirements in the specific visual worlds of animals. It goes without saying that this can only be achieved by respecting the ethological setting, and the particular viewpoint of specific animals (cf. Zeil and Zanker 1997; Smolka and Hemmi 2009; Zimmermann et al. 2018; Baden et al. 2020; Bergman et al. 2021; Nilsson and Smolka 2021; Qiu et al. 2021).

**Fig. 1:**
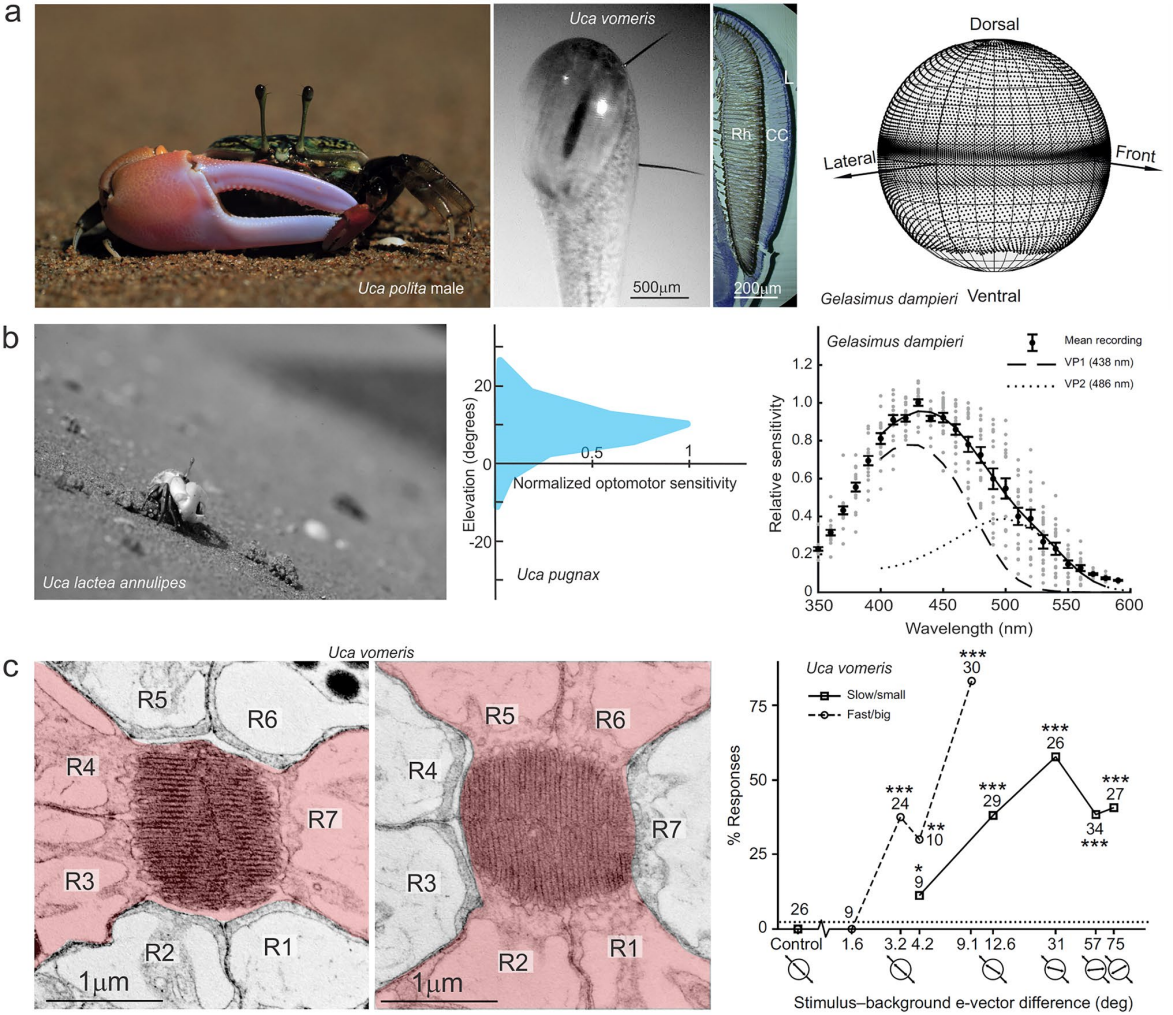
The visual system of fiddler crabs. **a** The crabs carry their eyes on long vertical stalks. The vertical eye radius is much larger than the horizontal eye radius, thus generating two vertical acute zones, the major one centred on the horizon. **b** The crabs align their eyes with the local visual horizon. The optomotor sensitivity to horizontal movement is restricted to a narrow, 20° wide band above the horizon with a maximum at 10^o^ elevation. Photoreceptor spectral sensitivity of the main retinular cells R1-R7 in *Gelasimus dampieri* peaks between 420 and 460 nm and there is evidence for additional UV sensitivity provided by retinular cell R8. **c** Microvilli directions in the main rhabdom are alternatingly orthogonal with horizontal directions in R3, R4 & R7 and vertical directions in R1, R2, R5 & R6. Behavioral experiments with fiddler crabs have documented high sensitivity to e-vector contrast. Figure credits: a: image 2 and 3 from left: modified from Alkaladi and Zeil (2014). a right: from Bagheri et al. (2020). b left: modified from Zeil and Al Mutairi (1996). b centre: after Kunze (1963). b right: modified after Jessop et al. (2020). c left and centre: modfied after Alkaladi and Zeil (2014). c right: from How et al. (2012)

To address in particular the problem of visual topography, I focus here on the description of a specific visual habitat, a tropical mudflat, which is both relatively simple and, as far as visual information content is concerned, highly structured. I describe this habitat from the viewpoint of fiddler crabs, arguably it’s most delightful inhabitants, because we know a fair amount about their visual system, their behavior, and the position and orientation of their eyes in space (Fig. 1; reviewed in Zeil and Hemmi 2006, 2014). The crabs live on the ground-plane of an essentially flat world and carry their eyes on long vertical stalks (Fig. 1a). The compound eyes of the crabs show specific adaptations to the conditions of vision in a flat world: each eye has a visual field covering the whole 360° panorama, and probably around 320° in elevation. Fiddler crabs possess a panoramic equatorial acute zone in which specifically vertical resolving power is increased, and which is aligned with the local visual horizon (Fig. 1a Zeil et al. 1986, 1989; Nalbach et al. 1989; Land and Layne 1995a; Zeil and Al-Mutairi 1996; Layne et al. 1997; Layne 1998; Smolka and Hemmi 2009; Bagheri et al. 2020). Fiddler crabs do not make target-directed saccadic or smooth pursuit eye movements, but the orientation of the eyes relative to the visual scene is controlled by visual—and possibly also mechanosensory-control systems around the three rotational axes (Fig. 1b, for review see Nalbach 1990). Eye orientation around the yaw axis is kept constant during locomotion, by powerful optomotor reflexes, except for fast resetting saccades after larger body turns (see Paul et al. 1990, 1998 for other crab species). As a consequence, the projection of the environment onto the eyes of a crab is predictable, and for three degrees of freedom also constant relative to an external coordinate system (height above ground, orientation in roll and pitch).

The ommatidia in fiddler crabs have the typical crustacean fused rhabdom with the horizontally oriented microvilli of retinular cells R3, R4 and R7 interdigitating with the vertically oriented microvilli of retinular cells R1, R2, R5 and R6 (Fig. 1c, Alkaladi 2008; Alkaladi et al. 2013; Alkaladi and Zeil 2014). A small retinular cell R8 with non-aligned microvilli directions sits in the light path distally to the main rhabdom. The main retinular cells R1 to R7 are thus polarization sensitive (Fig. 1c, How et al. 2012, 2014, 2015) and have a broad blue-green spectral sensitivity (Fig. 1b, Horch et al. 2002; Jordão et al. 2007; Jessop et al. 2020). An additional UV sensitivity is likely to be provided by R8 (Jessop et al. 2020).

The visual world of fiddler crabs can thus be roughly described as follows: because they carry their eyes on long, vertically oriented stalks, adult crabs see the bodies of conspecifics always below the line of their visual horizon. Everything larger or higher than the eye height of a crab appears above the line of horizon, including flying and running predatory birds, and the claws of waving conspecifics. The line of horizon is thus a robust discriminator between predators and other crabs (Zeil et al. 1989; Land and Layne 1995a; Layne et al. 1997; Layne 1998; Hemmi and Zeil 2005; Hemmi 2005a, b). This particular situation thus allows us to address the ‘topography of vision’ (Hughes 1977) in fiddler crabs in a quantitative fashion, including the spatial distribution of light on the retina, the differences in spatial, spectral, and polarization characteristics in different parts of the visual field, and the biologically relevant information content of the visual environment.

## Materials and methods

### Recording

I used a compact spectrographic imager (casi-Compact Airborne Spectrographic Imager, Itres Research, Canada) to record 360° horizontal panoramas in an open tropical mudflat at Cungulla, Queensland, Australia (approximately 19° 25.6′ latitude, 147° 6.9′ longitude), in the Bowling Green National Park, south of Townsville. The casi is a pushbroom imager and was mounted approximately 20 cm above ground on a motorized turntable, which was carefully leveled. The assembly of turntable, power supply, instrument control unit, and connecting cables allowed scans of 220° in azimuth at any one time, which took about 10 to 15 min each. The imager was equipped either with a lens that had an 80.87° field of view (FOV), or with a 37.8° FOV lens fitted with a polarizer, which could be rotated in 30° steps. Integration times and turntable rotation speeds were adjusted to generate square pixels across and along the scan direction, resulting in a resolution of 0.158° with the 80.87° FOV lens or of 0.071° with the 37.8° FOV lens. Spectral resolution varied between 8 and 12 nm, over a wavelength range between 418 and 963 nm, or between 380 and 920 nm. Recordings were done in October 1997 and September 1999.

### Calibration and data analysis

The casi is a CCD-based imager, in which the image of a scene is focused by the objective lens onto a slit. Light exiting from the slit is collimated and meets a reflection diffraction grating. The spectrally dispersed light is refocused onto a CCD, such that consecutive 2 nm wide spectral bands are imaged onto neighbouring CCD rows. The casi is calibrated in several steps, using a luminance standard (Model LS-65-8D Rev. B, Hofmann Engineering Corporation, Stamford, USA), and a suite of spectrum lamps (Helium, Hydrogen, Mercury and Oxygen). The calibration involves tests for accurate alignment and positions of spectral bands on the CCD, the determination of noise floor and system gain, the correction for pixel-to-pixel variations in the CCD, for vignetting, for slit irregularities, for spectral transmittance, and for entrance port reflections. The calibration results in Radiant Sensitivity Coefficient matrices, which contain the calibration values for each CCD pixel.

The raw data were stored on tape, and resulted after radiometrical calibration in a series of single-waveband images with pixel values in units of µW sr^−1^ cm^−2^ nm^−1^ s^−1^ (Spectral Radiation Units, SRU). Data consisted of digital numbers (DN: 0 < DN < 32,767) with 1 SRU = 500DN.

We converted SRUs to photons sr^−1^ cm^−2^ nm^−1^ s^−1^:

1 $${{SRU} \mathord{\left/ {\vphantom {{SRU} {e(\lambda )}}} \right. \kern-\nulldelimiterspace} {e(\lambda )}}\, = \,{\raise0.7ex\hbox{${DN}$} \!\mathord{\left/ {\vphantom {{DN} {(500e(\lambda ))}}}\right.\kern-\nulldelimiterspace} \!\lower0.7ex\hbox{${(500e(\lambda ))}$}}\, = \,DN\,\lambda \,1.0055\,10^{7}$$

with energy of 1 photon: e(λ) = 1.989 10^–10^ / λ [µW] (λ in units of [nm])

and, since we are concerned with vision, I transformed the images subsequently to units of photons pixel^−1^ s^−1^:

2 $$DN\,\lambda \,1.0055\,10^{7} \,(3.38\,10^{{ - 6}} )\,g1\,rowsum\,2\pi \,(1 - \cos ({\raise0.7ex\hbox{$1$} \!\mathord{\left/ {\vphantom {1 {2f\# }}}\right.\kern-\nulldelimiterspace} \!\lower0.7ex\hbox{${2f\# }$}}))$$

= photons sr^−1^ cm^−2^ nm^−1^ s^−1^ * pixelarea * spectral bandwidth * fraction of solid angle with g1 = the spectral bandwidth of one spectral pixel; rowsum = spectral bandwidth (number of spectral pixels summed); f# = f-number. The pixel solid angle is given by pixelarea/f^2^ = 3.38*10^− 6^/f^2^ [sr] with f = focal length, the conversion of solid angle to square degrees is 1 sr = 3.283*10^3^ [deg^2^] and pixel square degree is (3.38*10^− 6^ * 3.283*10^3^)/f^2^ = 0.0111/f^2^ [deg^2^/pixel], thus:

3 $$photons\,{\mkern 1mu} \deg ^{2} {\mkern 1mu} s^{{ - 1}} = DN * \lambda * g1 * {\mkern 1mu} rowsum * f^{2} * 2\pi * (1 - \cos ({1 \mathord{\left/ {\vphantom {1 {2f\# }}} \right. \kern-\nulldelimiterspace} {2f\# }})) * 3.061{\mkern 1mu} 10^{3}$$

Down-stream processing was done with ENVI software (L3Harris Geospatial Inc) and Matlab (The MathWorks Inc., Nattick, USA). I follow Nilsson and Smolka (2021) in showing vertical and horizontal transects through the scenes, the spectral composition of the sky and the ground and of other features in the scene as medians, 25th and 75th percentiles for wavelength bands 380–500, 500–600, 600–700 and 380–700 nm. Pixel values in image regions close to the sun in which the imager was saturated were replaced by NaNs (‘not a number’) for all analyses. No attempt was being made to determine degree and angle of polarization, because scans through different polarizer settings were not synchronous in time and also not perfectly aligned on a pixel-by-pixel resolution. I used sampling array data from Bagheri et al. (2020) and spectral sensitivity data from Jessop et al. (2020) to illustrate how mudflat scenes might look like through the compound eye array and the main spectral sensitivity of fiddler crabs.

### Image statistics

Spatial frequency power spectra at different wavelengths were determined for selected image regions using Matlab’s two-dimensional fast fourier function fft2 after applying a Hannning window and autocorrelation functions using Matlab’s inverse fast fourier transform ifft2.

### Spectral reflectance measurements

Spectral reflectance measurements of fiddler crab claw colours were made with an Ocean Optics USB4000 fibre optics spectrograph with the probe at 90^o^ to the illumination with a Xenon light source, so that both probe and illumination fibres were directed at an angle of 45^o^ relative to the normal of the measured surface.

## Results

### The vertical distribution of light

**Fig. 2:**
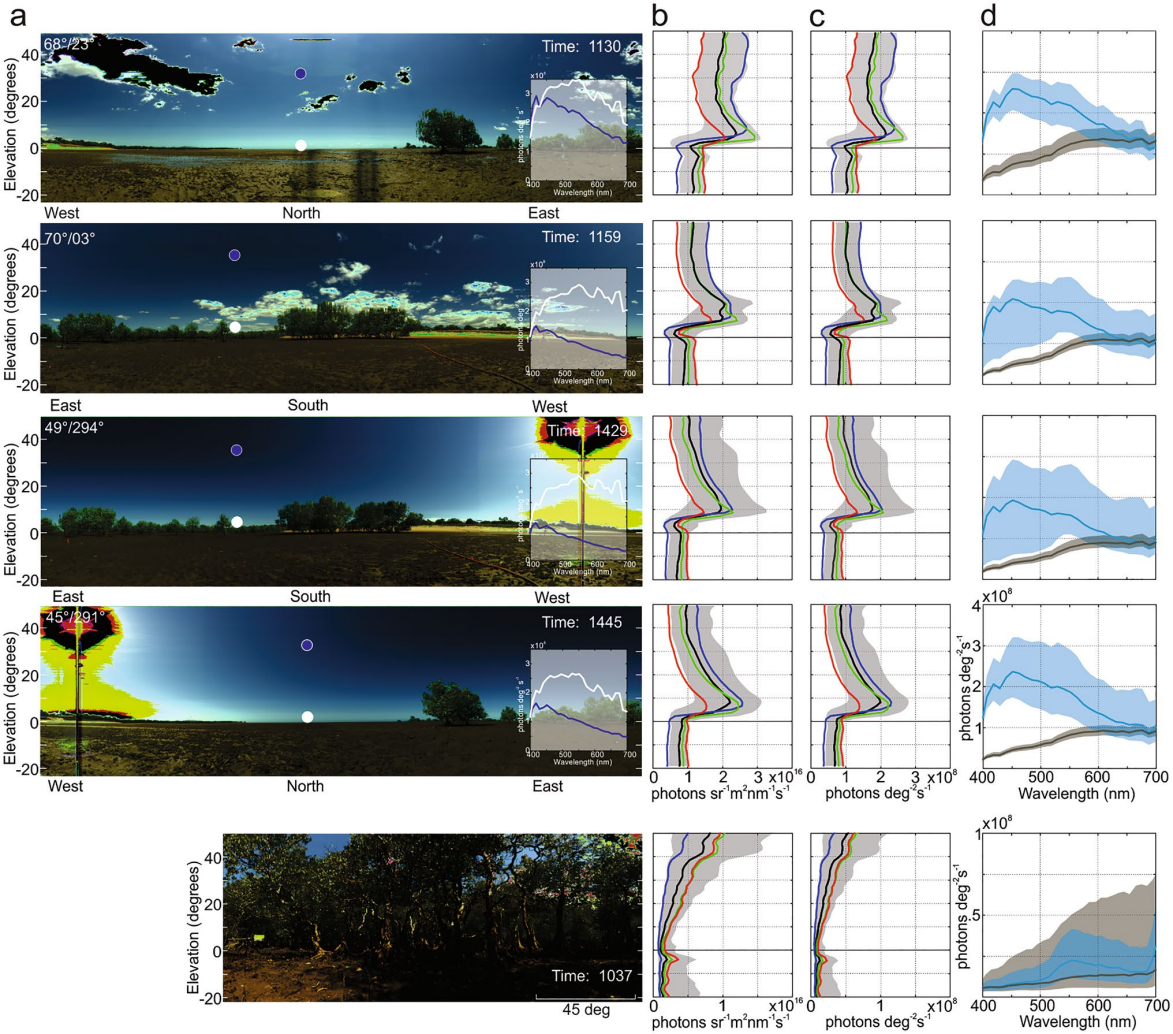
The vertical distribution of light in a tropical mudflat.** a** RGB images show spectrographic imager scans in different compass directions at different times of day. Insets show sky spectra close to the horizon (white) and at about 30^o^ elevation (dark blue) at the locations indicated by equivalently colored circles. Sun position (elevation/azimuth) are given in the top left corner of the scans (from https://geodesyapps.ga.gov.au/azimuth). Bottom scan is from inside a stand of mangroves for comparison. **b** Vertical distribution of median radiance in units of photons sr^−1^ m^2^ nm^−1^ s^−1^ at 380–500 nm (blue), 500–600 nm (green) and 600–700 nm (red). Black curve and grey shaded area show median radiance across the spectrum (380-700 nm) with 25th and 75th percentile, respectively. **c** Same with radiance in units of photons deg^−2^ s^−1^. **d** Median spectral composition of the sky (blue) and the ground (black). Shaded areas mark 25th and 75th percentile

The distribution of light intensity and spectral composition in a mudflat is not uniform. It changes with azimuth, with time of day and with cloud cover, as demonstrated by successive scans into northerly directions at 11:30 and 14:45 and into southerly directions at 11:59 and 14:29 in Fig. 2a. As the most striking observation I note that at least in the clear atmosphere of Australia there is a strong vertical gradient of skylight radiance, which is most clearly seen at medium (500–600 nm) and short wavelengths (380–500 nm) in the vertical distribution of light (Fig. 2b,c). This gradient is accompanied by a pronounced change in spectral composition (Fig. 2a insets) and reveals one of the most interesting facts of atmospheric physics (Lynch and Livingston 1995). Away from the horizon the atmosphere is optically thin, sunlight is affected by single scattering and the sky is blue (dark blue lines, insets Fig. 2a). Close to the horizon, the atmosphere is optically thick, and the spectrum is determined by multiple scattering, through which skylight approaches the spectrum of direct sunlight (white lines, insets Fig. 2a). Horizon light, in a narrow band of 5° above the horizon, is therefore white and, compared to the sun, forms with an angular extent of 1797 square degrees (cos(1^o^–5^o^) × 360^o^) the largest illuminant on earth (Lynch and Livingston 1995). Overall, the difference in spectral composition of skylight and light reflected from the substratum is highest at wavelengths between 380 and 600 nm (Fig. 2d). There is little contrast between sky and the ground at wavelengths above 600 nm.

The vertical distribution of light does depend on the position of the sun and on viewing direction, with the horizon discontinuity becoming more pronounced at lower elevations of the sun (compare scans at different times of day in Fig. 2).

The bottom row in Fig. 2 shows for comparison a scan inside a dense stand of mangroves where there is no horizon discontinuity in the vertical distribution of light and no distinct difference between sky and ground due to a rich cutter of vegetation, patches of skylight and shadows.

### The distribution of polarized light

Fiddler crabs view the world through photoreceptors with high polarization sensitivity because their microvilli are aligned in vertical and horizontal directions (Alkaladi et al. 2013; Alkaladi and Zeil 2014). It is, therefore, of interest to investigate the distribution of polarized light in their habitat. Given that the absorption properties in fiddler crab rhabdoms are not clearly understood and are also likely to vary across the visual field (see Alkaladi et al. 2013), it seems premature to try to link the distribution of light to photoreceptor activation. Instead, I simply present in what follows the distribution of horizontally and vertically polarized light to characterize differences in the input to the orthogonal polarization sensitivities in different parts of the visual field. These differences in the light field may be helpful in future to understand the functional significance of microvilli banding patterns and of down-stream processing of the two classes of polarization sensitivities, once they become known in more detail.

**Fig. 3:**
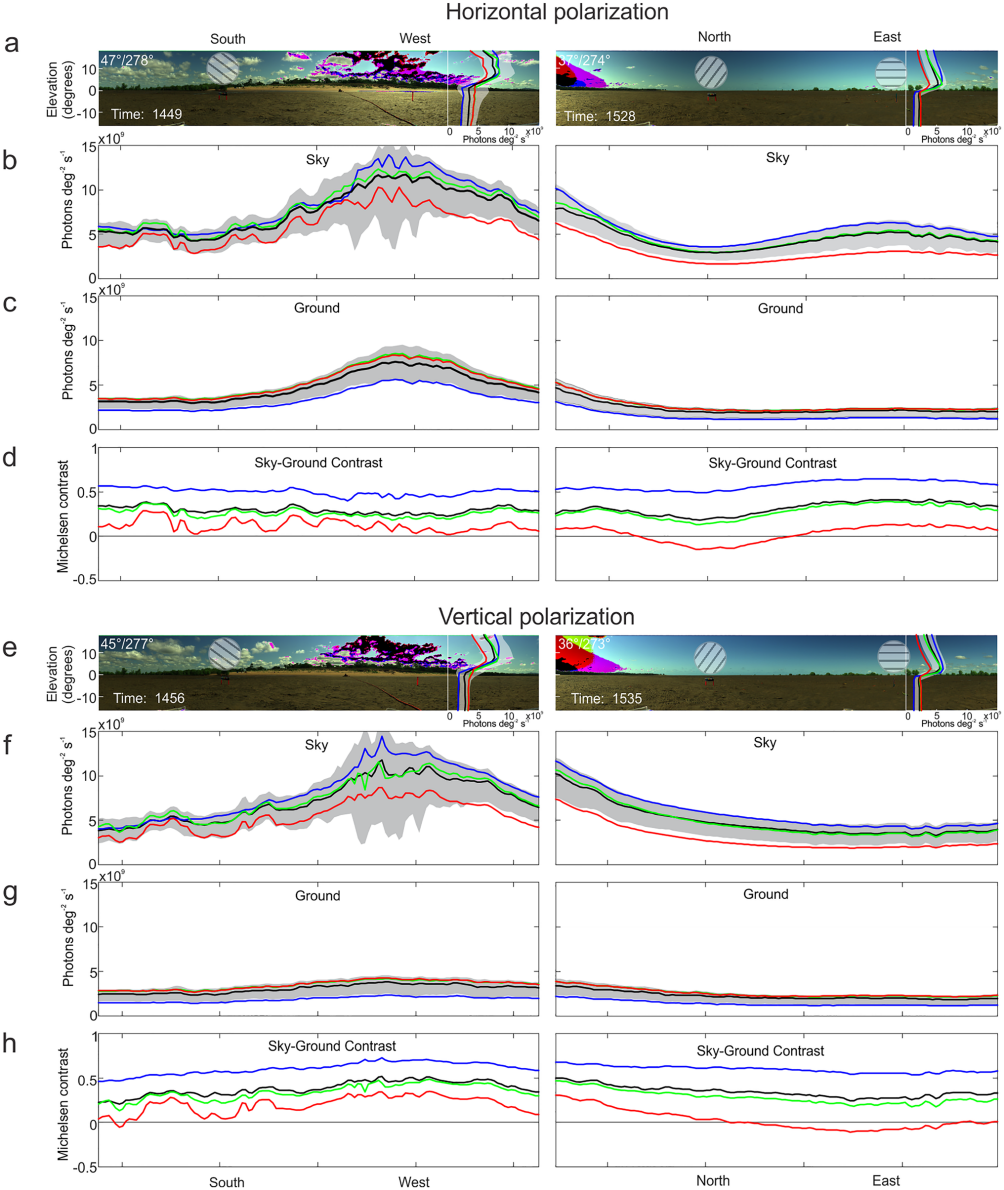
The distribution of light in a tropical mudflat. **a** Panoramic RGB images of slightly overlapping South-West and North-East spectrographic imager scans recorded through a horizontal polarizer. Black image regions indicating imager saturation, where replaced by NaNs for analysis. Sun position (elevation/azimuth) is given in the top left corner of the scans. Insets show medians of vertical distribution across the scans in wavelength ranges as indicated below. Vertical extent of scans: 37.8^o^. **b and c** Median radiances along 3^o^ vertical slices through the celestial (**b**) and the terrestrial part (**c**) of the scans in wavelength bands 380–500 nm (blue), 500–600 nm (green), 600–700 nm (red) and 350–700 nm (black). Grey shaded area marks 25th and 75th percentile of the 350–700 nm wavelength range. **d** Michelsen contrast between sky and ground in the same wavelength ranges. **e to h** Same for scans recorded through a vertical polarizer

Skylight intensity and the amount of light reflected from the surface are much higher in the sector containing the sun than in other directions of view (Fig. 3a–c). Both skylight and light reflected from the ground are polarized (compare Fig. 3a–c with Fig. 3e–g). At the time of day this particular panorama was recorded (early afternoon, between 14:45 and 15:35 local time), the intensity of horizontally polarized light begins to decline at right angles to the sun in the North and in the South. In these compass directions, the band of maximal polarization, which is found on a great circle 90^o^ away from the sun, perpendicular to the solar meridian, intersects the horizon.

Light is polarized parallel to the great circle and, as the sun sinks, the band of maximal polarization pivots around an axis perpendicular to the solar meridian so that the sky viewed along this axis which is approximately oriented in a north–south direction becomes increasingly vertically polarized. With the sun in the zenith, the band of maximal polarization lies on the horizon and light is horizontally polarized. Note that viewed through a horizontal polarizer, the sky is bright opposite the sun in the East (right image, Fig. 3a), because skylight is horizontally polarized along the solar ephemeris. For a more quantitative appreciation of these azimuth variations in skylight intensity I plot the horizontal distribution of skylight and the substratum through a horizontal (Fig. 3b, c) and a vertical polarizer (Fig. 3f, g) at the four different wavelength bands, 380–500 nm (blue), 500–600 nm (green), 600–700 nm (red) and 380–700 (black). These horizontal distributions show clearly that the sky is not a homogeneous visual background. Superimposed on the smooth azimuth gradient of skylight intensity is the large clutter of clouds, which reflect and scatter approximately double the amount of light than the celestial background (see below).

Light reflected from the ground in general follows the azimuth gradient of skylight luminance, although at much lower absolute intensities (Fig. 3c and g). Interestingly, not only the mean intensity of light reflected from the ground is dependent on both azimuth and wavelength, but also the ‘clutter’ generated by shadows and specular reflections (see below). Throughout the panorama, the sky-substratum contrast is highest at short wavelengths and for vertically polarized light (Fig. 3d and h). Several factors contribute to this effect. The azimuth variation in light intensity is similar for skylight and for light reflected from the ground, the reflections from the substratum are horizontally polarized, and the ground generally returns less short-wavelength light, but is highly reflective at long wavelengths, while the situation is reversed for the sky.

### The spectral distribution of polarized light

**Fig. 4:**
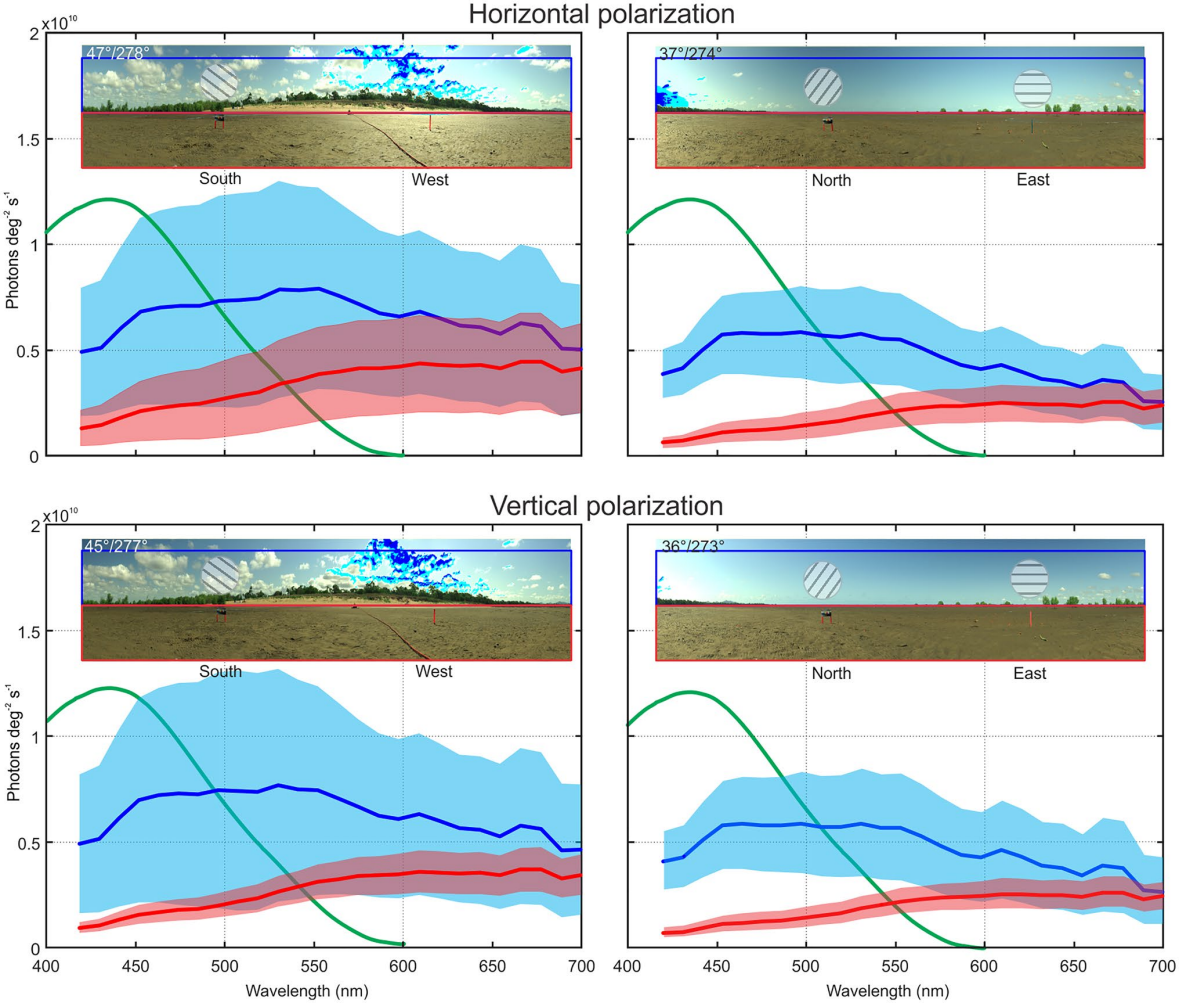
Spectral and polarization differences between the sky and the ground in different compass directions. Top panels through a horizontal polarizer, bottom panels through a vertical polarizer. Graphs show the medians (thick lines) and 25th and 75th percentiles (shaded areas) of photons deg^−2^ s^−1^ over wavelength for the sky from the horizon to 15^o^ elevation (blue) and for the ground from the horizon to 15^o^ below the horizon (red). Thick green line is the average spectral sensitivity of fiddler crab photoreceptors as determined by intracellular recordings (from Jessop et al. 2020)

The spectral characteristics of both skylight and reflected light from the ground vary with azimuth and the direction of polarization (Fig. 4). In these early afternoon scans, the sky begins to brighten in the South-West and skylight variance is increased by clouds (left panels, Fig. 4), compared to the situation facing North-East (right panels, Fig. 4). Specular reflections and shadows on the ground make the surface in the West brighter and reflectance more variable (top left panel, Fig. 4). Because specular reflections are horizontally polarized, this variance is reduced when looking through a vertical polarizer (bottom left panel, Fig. 4). Overall, skylight and surface reflections are less variable across the spectrum in viewing directions away from the sun (right panels, Fig. 4). As documented before, sky-ground contrast is highest in the short-wavelength part of the spectrum and the spectral sensitivity of the retinular cells contributing to the main rhabdom covers that part of the spectrum (green line in Fig. 4).

### Clouds, specular reflections and shadows

**Fig. 5:**
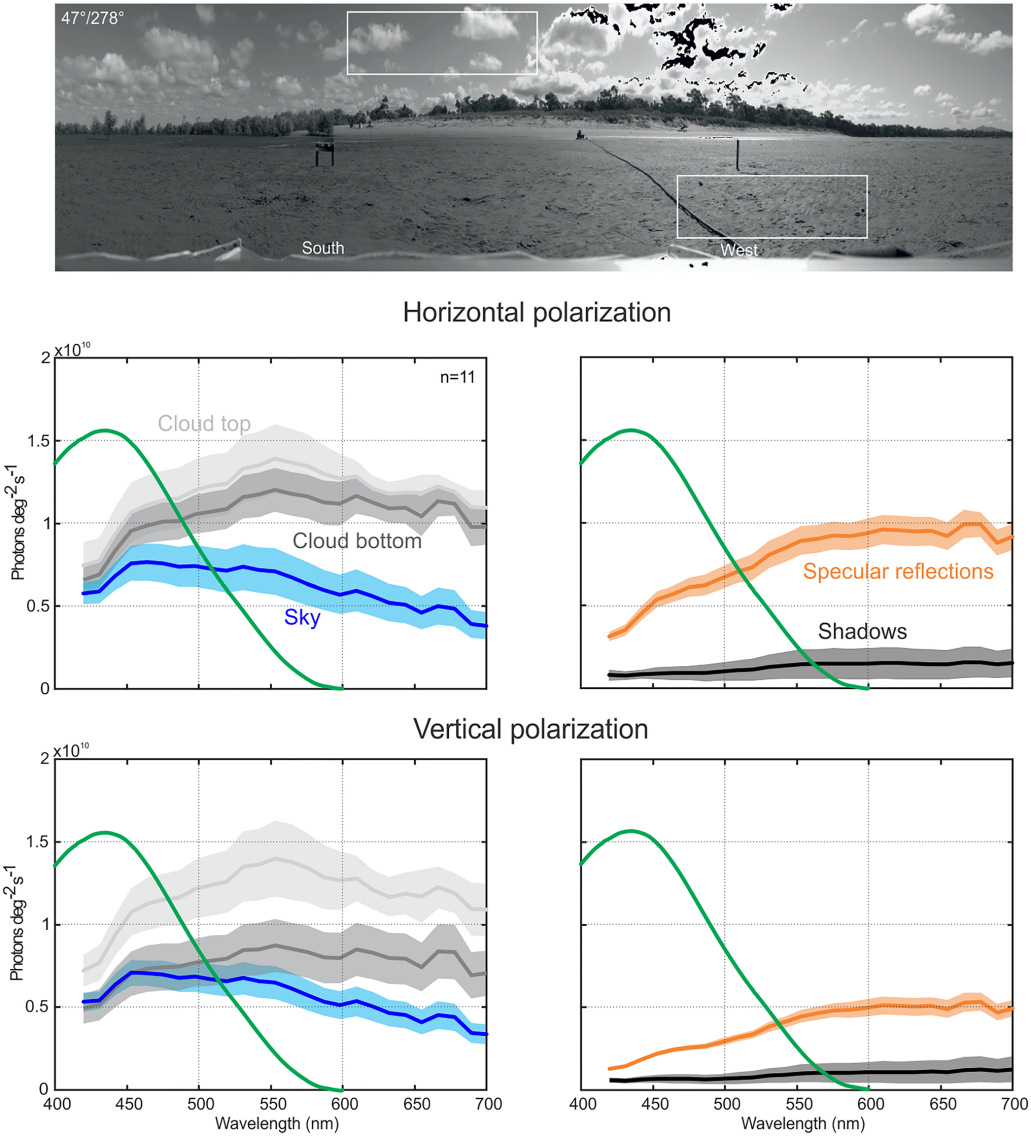
Sky and ground clutter. At 11 positions each, spectra were collected by eye in sky and ground areas (as indicated by white rectangles in the scan shown on top) of cloud tops, cloud bottoms and areas of clear sky from scans through a horizontal and vertical polarizer (top and bottom diagrams on the left, respectively). On the ground (diagram on the right) spectra were collected from 11 points with specular reflections and 11 points in shadow. Thick green line indicates the spectral sensitivity of fiddler crab main retinular cells as measured intracellularly by Jessop et al. 2020. Solid lines show means of 11 measurements and shaded areas standard deviation

The sky is not a uniform background for vision. As we have seen before, luminance, spectral composition and polarization all vary systematically with azimuth and elevation. The distribution of light in the sky depends heavily on the position of the sun. In addition, the sky also acquires a more unpredictable structure through the presence of clouds (Fig. 5). The spectral composition of light differs between the open sky background and clouds and the cloud-sky contrast is therefore dependent on wavelength (left panels, Fig. 5). Because skylight is blue and because the sky is therefore rather dark at long wavelengths, clouds are much more dominant features in the sky when viewed at long compared to short wavelengths. Cloud-sky contrast consequently is smallest in the short-wavelength range covered by the spectral sensitivity of the fiddler crab main rhabdom (green line in left panels, Fig. 5).

Ground reflections are highest in westerly directions towards the sun and decrease with azimuth distance from the sun (Fig. 3c) and are strongly horizontally polarized (compare Fig. 3c g), suggesting that its source is a mixture of specular reflections and shadows, generated by a corrugated, wet, and sandy terrain. Specular reflections are strongly polarized, to the degree that their intensity is more than halved throughout the spectrum when viewed through a vertical polarizer (right panels, Fig. 5). The spectral signatures of shadows are only weakly polarized. When viewed in north-easterly directions, away from the direction of illumination in this early afternoon scene, the substratum appears much more uniform (Figs. 3 and 4). The reason being, that there are practically no shadows and few specular reflections on the substratum when viewed from the direction of the sun.

### The spatial statistics of mudflat scenes and the fiddler crab sampling array

**Fig. 6:**
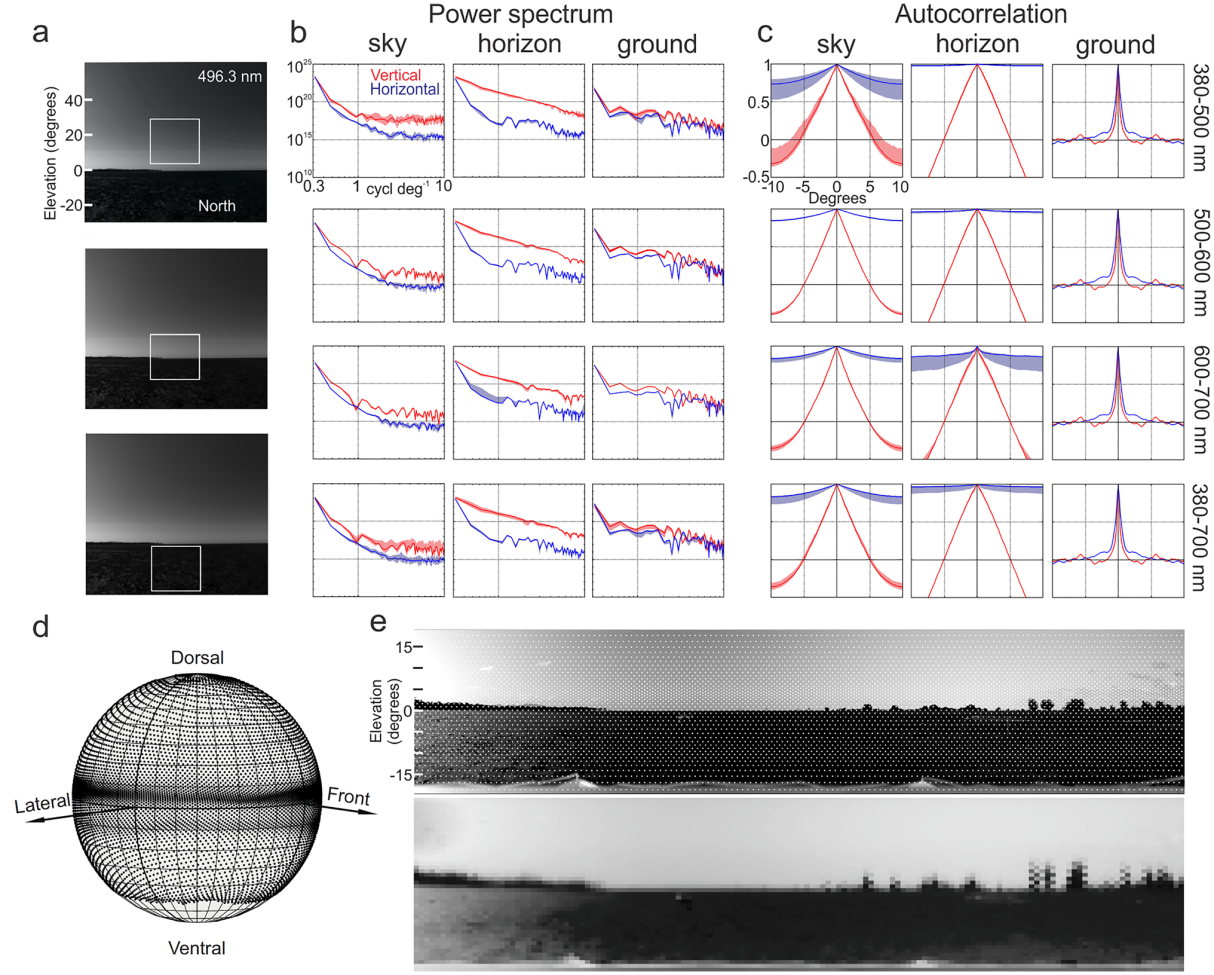
Spatial statistics of the mudflat scene. **a** White squares show 20^o^x20^o^ images segments above, on and below the horizon facing north for which average power spectra and autocorrelation functions were determined across different wavelength ranges. **b** Power spectra in vertical (red) and horizontal direction (blue) for sky, horizon and ground image segments for wavelength ranges 380–500 nm (1st row), 500–600 nm (2nd row), 600–700 nm (3rd row) and 380–700 nm (4th row). Shown are medians (solid lines) and 25th and 75th percentiles (shaded areas). **c** Same for autocorrelation functions. **d** The sampling array of *Gelasimus dampieri* as determined by Bagheri et al. (2020). **e** The mudflat facing north with superimposed sampling array (top) and filtered through the sampling array (bottom)

Does the fiddler crab sampling array, as recently most accurately determined by Bagheri et al. (2020), reflect the spatial structure of mudflat scenes? To address this question, I determined the power spectrum and the autocorrelation for three 20^o ^× 20^o^ wide image segments (Fig. 6a) above, at and below the horizon in a northerly direction where this particular mudflat scene is least obstructed by vegetation. The distributions of power spectra and autocorrelations in vertical (red) and horizontal directions (blue) for wavelength ranges 380–500 nm, 500–600 nm, 600–700 nm and 380–700 nm are shown in Fig. 6b, c. There is consistently more power at spatial frequencies between 0.5 and 10 cycles/degree in vertical compared to horizontal directions in the sky segment and in particular on the horizon and at short wavelengths (380–500 nm). At medium and long wavelengths, there is more power at higher frequencies on the ground, compared to the sky (compare sky and ground spectra, Fig. 6b). The autocorrelation function is consistently broader in the sky, compared to the ground at all wavelengths and much more so in horizontal, compared to vertical directions (Fig. 6c).

If the sampling array of crabs would reflect these image statistics, it would show higher resolution in the ventral, compared with the dorsal visual field and overall, higher vertical, compared to horizontal resolution (both sampling resolution (interommatidial angles) and optical resolution (acceptance functions). Instead, horizontal resolution is fairly constant across elevation, while vertical resolution is locally sharply increased in a band 5^o^ above the horizon and 10^o^ below the horizon (Fig. 6d and e; see Bagheri et al. 2020). Otherwise the crab eye under-samples the visual scene both in the dorsal and the ventral visual field (Fig. 6d and e), with little variation in the size of acceptance functions, because rhabdom diameters (Alkaladi and Zeil 2014) and facet lens diameters do not vary much throughout the eye, except for the most dorsal and ventral eye regions (Zeil and Al-Mutairi 1996; Smolka and Hemmi 2009; Bagheri et al. 2020).

### The mudflat through fiddler crab eyes

**Fig. 7:**
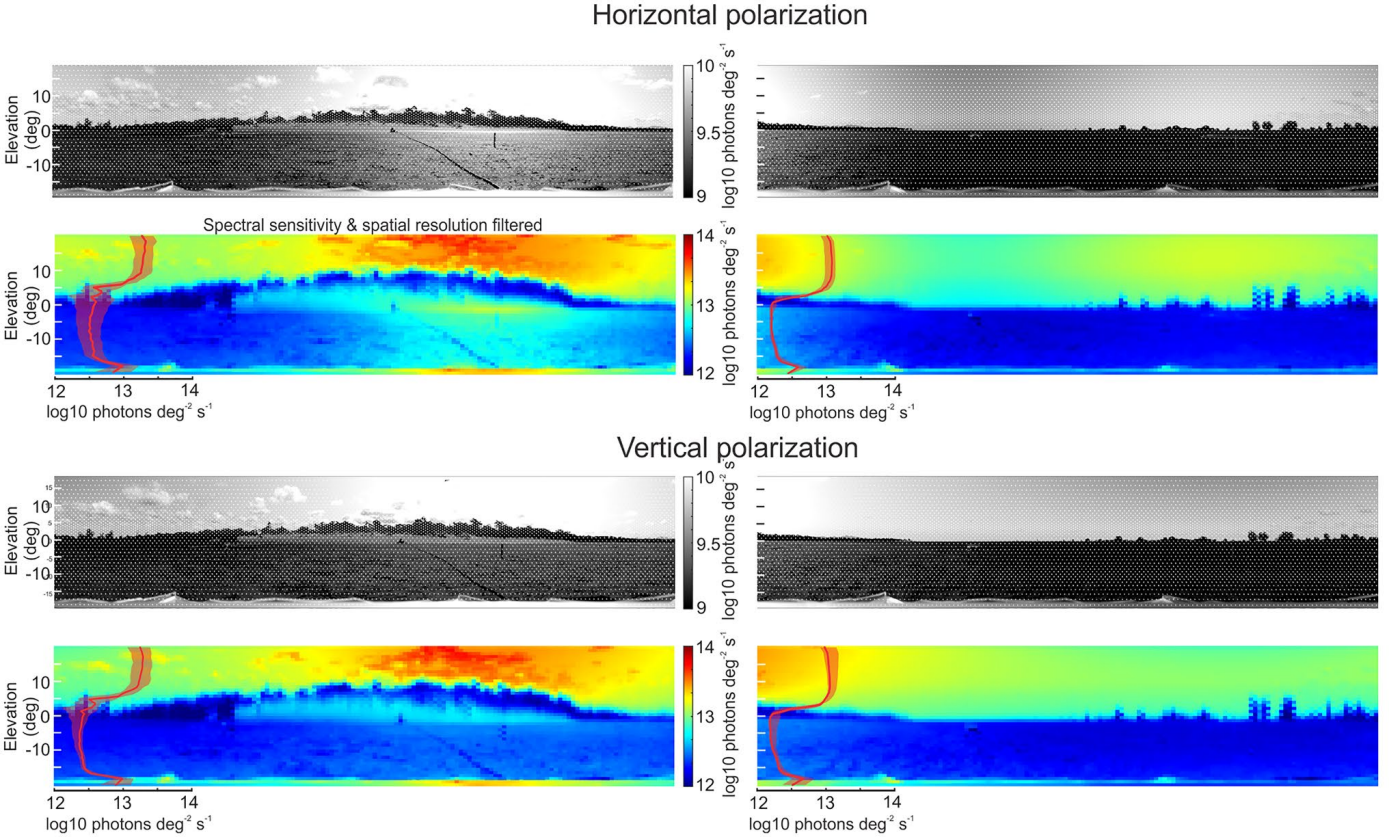
The mudflat scene through fiddler crab eyes. Top panels horizontally, bottom panels vertically polarized light. Grey panels show the scene at 450 nm with the sampling array as determined by Bagheri et al. 2020 superimposed as white dots. False color images show the same scene filtered by the sampling array, the optical resolution (at full width at half maximum of the Airy disk (Bagheri et al. 2020) and by the blue-green spectral sensitivity of the fiddler crab eye (Jessop et al. 2020). Inset graphs show the median and 25th and 75th percentile of the vertical distribution of light across the whole scans

To get a first impression on how the mudflat may be seen by fiddler crab eyes, I sampled the radiance across the spectrum at locations defined by the crabs’ sampling array, that is the viewing directions of individual ommatidia as determined by Bagheri et al. 2020 (white dot array in grey-level images, Fig. 7). I also took account of the slightly varying optical resolution across the visual field by integrating photon counts over the angular size of the Airy disk using data from Bagheri et al. 2020. I finally filtered the spatially sampled, integrated spectral radiance with the spectral sensitivity of the crabs’ main retinular cells using the intracellular measurements by Jessop et al. 2020. The results are shown as false color images of log10 photons deg^-2^ s^-1^ for both vertically and horizontally polarized light in Fig. 7.

I note first, the magnification of the horizon line, which is due to the large increase of vertical resolution in the horizontal visual streak of fiddler crab compound eyes (Land and Layne 1995a; Zeil and Al-Mutairi 1996; Smolka and Hemmi 2009; Bagheri et al. 2020); second, the large difference between sky and ground radiance (see vertical profile insets, Fig. 7) and thirdly, the reduction of visual clutter both in the sky and on the ground, which is due to spatial low-pass filtering and spectral sensitivity being restricted to the short-wavelength part of the spectrum. I add the caveat that the scans do not cover the spectrum below 380 nm, which the crabs’ spectral sensitivity does and that the filtering I employed has not taken account of the diameter, the varying length and banding patterns, nor the screening pigments of fiddler crab rhabdoms and the way in which they affect photon absorption (see Alkaladi et al. 2013; Alkaladi and Zeil 2014; Jessop et al. 2020).

## Discussion

### Natural scenes

The mudflat environment is a particularly clear case of the predictable spatial structure of the distribution of environmental light, as described recently for many terrestrial habitats by Nilsson and Smolka (2021). The sky is brightest and whitest close to the horizon and because the ground has a low reflectance at short wavelengths, the highest sky-substratum contrast is in the short-wavelength region of the spectrum. Sky-substratum contrast can be reversed at long wavelengths. There are clear differences in sky-substratum contrast when the horizon is viewed through a vertical or a horizontal polarizer, most prominently in the direction of the sun. The sky-substratum differences in spectral and spatial dimensions are a constant feature of the open mudflat environment, as are the vertical and horizontal gradients of skylight intensity and spectral composition. Both sky-substratum differences and skylight gradients change predictably with sun position, but are subject to large variations due to the presence of clouds. Sun position also predicts the gradients of spatial, wavelength-dependent clutter on the ground.

Past studies of the statistics of natural scenes have noted large variations in the details of spatial statistics (e.g. van Hateren and van der Schaaf 1996), but it has apparently not been realized, that these variations are not uniform across the visual field and are systematic and predictable across the horizon line separating the celestial and terrestrial hemispheres (e.g. Atick 1992; Ruderman 1994; Field 1987). Students of the diversity of visual systems, in contrast, have repeatedly noted functional differences in photoreceptor spacing and spectral sensitivities between the dorsal and the ventral visual field in many animals (e.g. Walls 1942; Hughes 1977; Zeil et al. 1996 Hemmi and Grünert 1999; Collin 1999; Land 1999a; Smolka and Hemmi 2009), which are now increasingly being described in detail as adaptations to specific tasks and to the specific distribution of environmental light (e.g. Zimmermann et al. 2018; Baden et al. 2020; Bergman et al. 2021; Qiu et al. 2021).

The way in which fiddler crab eyes sample the world does reflect some spatial properties of mudflat scenes, but not in the ‘classical’ ways of optimal sampling, where optical resolution (the size of the acceptance function Δρ) is matched to the sampling array (the interommatidial angles Δφ) so that Δρ = 2Δφ (e.g. Warrant and McIntyre 1993), or where overall resolution and receptive fields are designed to optimally represent the spatial statistics of natural scenes (e.g. Field 1987; Srinivasan et al. 1982). Optical resolution in fiddler crab eyes is only matched to the sampling array within the narrow bands of highest vertical resolution (Zeil and Al-Mutairi 1996; Smolka and Hemmi 2009), which lie above and below the horizon in *Gelasimus dampieri* and above and on the horizon in *Tubuca flammula* (Bagheri et al. 2020). In the rest of the eye, acceptance functions are much smaller than interommatidial angles (Zeil and Al-Mutairi 1996; Smolka and Hemmi 2009; Bagheri et al. 2020). The sampling array does also not reflect the spatial statistics of mudflat scenes, which would require much higher resolution in the ventral compared to the dorsal visual field, as judged by the differences in power spectra and in the width of the autocorrelation function (Fig. 6). Even if there was selective pressure in fiddler crabs to match resolution to spatial statistics, there are serious constraints on size and shape of compound eyes, because their sampling array is largely determined by the local eye radius R, with Δφ = A/R (A: facet lens diameter; see Land 1999a).

However, eye design in fiddler crabs, including their eyes being carried on long vertical stalks, does reflect other spatial properties of the mudflat environment, most importantly the fact that it is a flat world with a prominent horizon line, which divides the visual scene into two distinct zones of biologically relevant information (Zeil et al. 1986; Layne et al. 1997; Layne 1998; Zeil and Hemmi 2006): the dorsal visual field viewing bird predators and the crabs’ claw waving signals and the ventral visual field which views the bodies of other crabs. In addition, elevation in the visual field provides information on distance in a flat world, information which may have shaped the gradients of vertical resolution in the visual streaks of fiddler crab eyes (Zeil et al. 1986; Smolka and Hemmi 2009; Bagheri et al. 2020).

### Environmental light in mudflats and the visual tasks of fiddler crabs

**Fig. 8:**
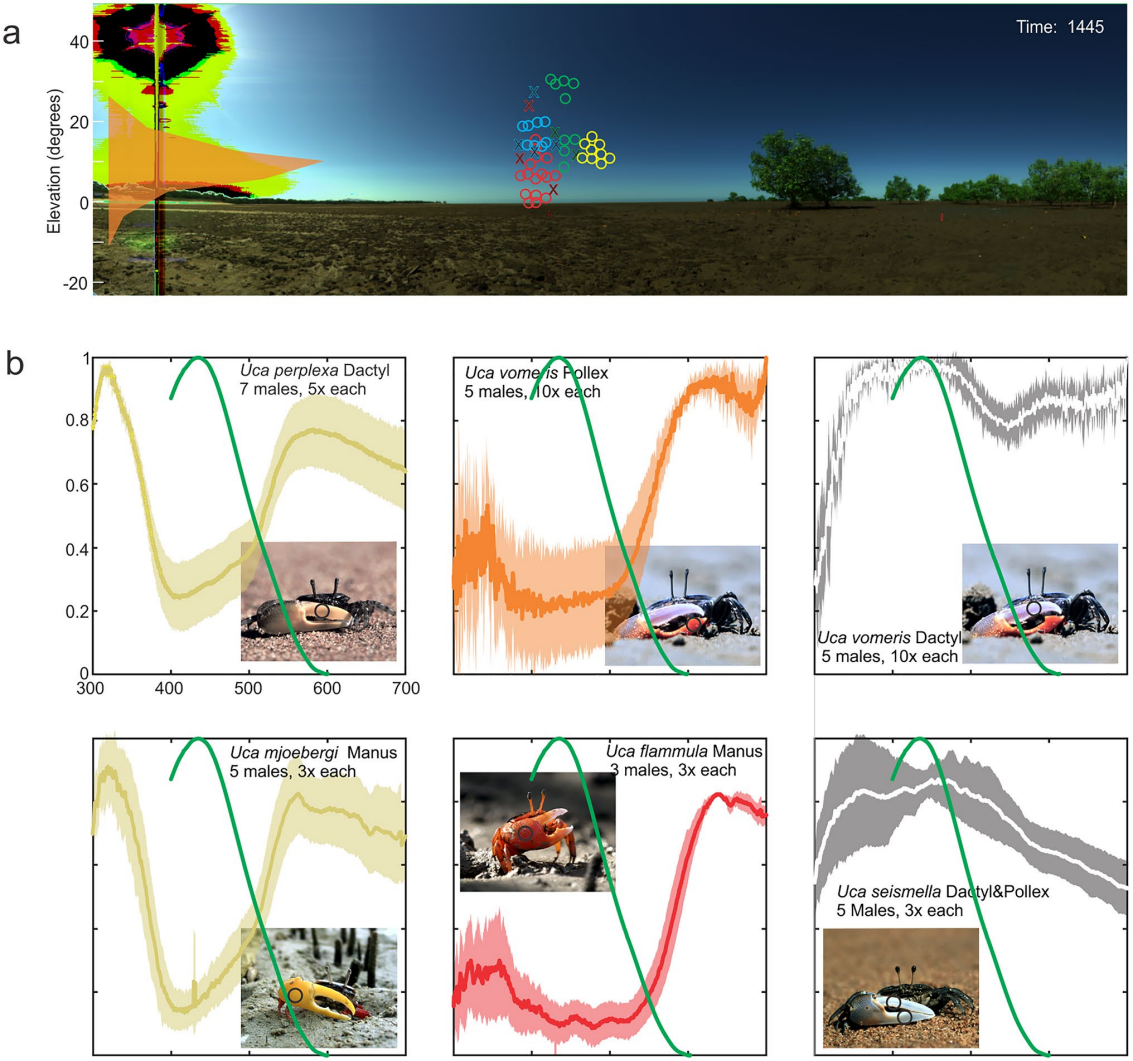
The mudflat scene as the background for fiddler crab visual tasks. **a** Optomotor sensitivity (orange, after Kunze 1963) and elevation positions of birds and insects to which crabs responded by running back to their burrows (circles) and by entering their burrows (crosses) superimposed on a mudflat scan. Response location data from Smolka et al. 2011. **b** Reflectance measurements (means and standard deviation) of the enlarged male claws of five different species of Australian fiddler crabs as indicated in the panels together with and by the blue-green spectral sensitivity of the fiddler crab eye (green line, from Jessop et al. 2020). Measurement locations are indicated by black circles in the crab photographs. Photograph of *Uca mjoebergi* courtesy of Tanya Detto

Against the backdrop of environmental light, as describerd here, fiddler crabs are known to be concerned with the following visual information: they respond with eye movements to rotations of a polarizer in the dorsal visual field (Korte 1965, 1966), a response most likely mediated by heavily modified dorsal-most ommatidia (Alkaladi and Zeil 2014) and which indicates that a celestial polarization compass may be involved in path integration (Zeil 1998; Zeil and Hemmi 2014). Optomotor sensitivity for gaze stabilization around the yaw axis is restricted to a narrow band above the horizon (Figs. 1b and 8a, Kunze 1963), which is a simple way of separating rotational and translational optic flow in a flat world because above horizon visual features tend to be far away and thus do not generate image motion during translation (Nalbach and Nalbach 1987). Optomotor sensitivity does not make use of polarization contrast (Drerup and How 2021), but the crabs are very sensitive to the polarization contrast of approaching objects (How et al. 2012, 2014, 2015). The eye equator is aligned around roll and pitch axes by a position servo to the local visual horizon (Zeil and Al Mutairi 1996; Layne et al. 1997; see also Nalbach et al. 1989) and for this it may be significant that the spectral sensitivity of the main retinular cells R1-R7 covers the wavelength range below 550 nm where sky-ground contrast is highest (Fig. 4) and where intensity variations in the sky (blue sky and clouds) and on the ground (specular reflections and shadows) are smallest (Fig. 5). These control systems assure that the processing of visual information in the dorsal visual field can be dedicated to the specific visual tasks of detecting predators (Fig. 8a, Layne 1998; Layne et al. 1997; Smolka et al. 2011, 2013; Donohue et al. 2022) and - just above the horizon-claw waving signals (e.g.Land and Layne 1995a; Zeil and Zanker 1997; How et al. 2007, 2008, 2009). The crabs’ main predators, at least in this particular mudflat, are terns scanning for burrow-less crabs on the surface while flying parallel to the ground (Land 1999b). Although the dorsal visual field in fiddler crabs is under-sampled, the detection of birds is aided by the increased contrast sensitivity to small objects provided by small acceptance functions (Smolka and Hemmi 2009) and by the high polarization contrast sensitivity based on the orthogonal microvilli directions in retinular cells R1, R2, R5 and R6 (vertical) and R3, R4 and R7 (horizontal) (Alkaladi et al. 2013; Alkaladi and Zeil 2014; How et al. 2012, 2014).

Processing in the ventral visual field involves detecting conspecifics (Zeil and Hofmann 2001), distinguishing males from females (Land and Layne 1995a,b) and judging the distances of other crabs (How et al. 2008), which, for instance, is required to assess their size (Backwell and Passmore 1996; Backwell et al. 2000). Resident crabs detect and respond to other crabs approaching their invisible burrow, a task that must be guided by information from the path integration system (Hemmi and Zeil 2003a, b, c). It further involves discriminating between conspecifics and other crabs, between conspecific males and females and between resident neighbours and crabs wandering over the mudflat in search of a burrow (Zeil and Layne 2002 Hemmi and Zeil 2003a, b, c; Detto et al. 2006). In the ventral visual field, concerned with such social information, the crabs also make use of polarization contrast to detect specular reflections from the wet cuticle of other crabs (How et al. 2015), whereby this contrast and the background clutter against which it is seen depends on viewing direction relative to the sun (Fig. 2). At this stage, it is only known for some species of fiddler crabs, that conspicuous claw and body colouration (e.g. Detto et al. 2008) is used in species and neighbour recognition (Detto et al. 2006) and in mate choice (Detto 2007; Detto and Backwell 2009). Some fiddler crab males actively guide mate searching females by herding them toward their burrows (How and Hemmi 2008). Finally, although fiddler crabs rely largely on path integration for homing, they do see the burrow entrance at close range and correct their path accordingly during escape runs (Murakami et al. 2018) and in some cases, males construct mud hoods close to their burrows, which they use as landmarks (Kim et al. 2010; Kim and Christy 2015).

There are a number of visual processing tasks we know little about and which are worth investigating. For instance, it remains unclear to what extent vision in the ventral visual field is involved in claw guidance during feeding and during the frequent and skilful fights, in which particularly male fiddler crabs engage in. Much is also to be learnt about color and polarization vision in fiddler crabs. Color vision has been shown to be involved in mate choice in *Uca mjoebergi* (Detto 2007), with the UV reflectance of the male claw being crucial (Fig. 8b, Detto and Backwell 2009). UV reflectance is indeed common in fiddler crab male claws, irrespective of their color in the human visible part of the spectrum (Fig. 8b). Given that the mudflat background reflects little short-wavelength light, the UV reflectance from claws provides high-contrast signals (Zeil and Hofmann 2001), while this is not the case for the long wavelength reflectances, where we see most of the inter-species color variation (Fig. 7b). Electrophysiological evidence so far has revealed two spectral sensitivities in fiddler crabs, a broad one most sensitive to wavelengths between 420 and 460 nm (Fig. 8b, Jessop et al. 2020) and one sensitive in the UV below 360 nm (Jessop et al. 2020). This combination of spectral sensitivities does not allow discrimination of the long wavelength reflectances yellow and red that are so common in male fiddler crab claws (Fig. 8b). The distribution of spectral sensitivities across the eye of fiddler crabs remains to be investigated, but an attractive and testable hypothesis would be that there is an additional, long wavelength spectral sensitivity exclusively located in the ventral visual field. Equally, the spectral and polarization sensitivities involved in predator detection and in the control of compound eye orientation still need to be identified.

And lastly, to end with a caveat and challenge: A number of Australian fiddler crab species such as *Uca flammula*, *Uca coarctata* and *Uca dussumieri* live closer to and deep in mangrove forests where both geometrical constraints and the ambient light environment are very different compared to the open mudflat environment (see bottom scan Fig. 2). This is also true for populations of some of the species normally found on open mudflats. Do they rely more on a dorsal light reflex to align their eyes in the absence of a high-contrast horizon line? How do they detect aerial predators in a dorsal visual field that is now cluttered with wind-driven vegetation? Would one expect specific changes to the physiological properties of their visual systems, similar to the circadian shifts in absolute light sensitivity, spectral sensitivity and temporal summation that have been documented in the largely day-active *Gelasimus dampieri* (Jessop et al. 2020; Brodrick et al. 2022)?

In conclusion, fiddler crabs are most concerned with detecting changes caused by moving objects against the environmental light background described here, and I hope by making the data on the distribution of light in mudflats available that they are useful in future studies of photoreceptor physiology and in modelling photoreceptor activation and adaptation patterns in response to biologically significant events.

## Data Availability

10.25911/wcxh-ga12.
